# A Retrospective Analysis of Vitamin D Levels in Hospitalized COVID-19 Patients With Suspected Pulmonary Embolism

**DOI:** 10.7759/cureus.41805

**Published:** 2023-07-13

**Authors:** Shaun V Charkowick, Constantine N Logothetis, Katherine Tsay, Aryanna Jordan, Catherine Hanna, Sherry Zhang, Emily Coughlin, Thomas A Weppelmann, Rahul Mhaskar, Asa Oxner

**Affiliations:** 1 College of Medicine, University of South Florida Health Morsani College of Medicine, Tampa, USA; 2 Department of Internal Medicine, University of South Florida Health Morsani College of Medicine, Tampa, USA; 3 Department of Medicine, Kaiser Permanente Oakland Medical Center, Oakland, USA; 4 Department of Ophthalmology, University of South Florida Health Morsani College of Medicine, Tampa, USA

**Keywords:** american thoracic society, area deprivation index, icu, vitamin d, deep vein thrombosis, pulmonary embolism, covid-19

## Abstract

Introduction

Despite using anti-coagulation therapy in hospitalized coronavirus disease 2019 (COVID-19) patients, they have high rates of pulmonary embolism (PE) and deep vein thrombosis (DVT). The main objective of this study was to evaluate the association between vitamin D deficiency and thrombotic events (defined as the occurrence of a new PE or DVT) in hospitalized COVID-19 patients.

Materials and Methods

This was a retrospective, cross-sectional study of 208 hospitalized COVID-19 patients who received a computed tomographic pulmonary angiography (CTPA) based on clinical suspicion of PE between January 1, 2020, and February 5, 2021. A <20 ng/mL serum vitamin D level was used to categorize vitamin D deficiency. Nonparametric tests and multivariate binary logistic regression were used to evaluate the association between serum vitamin D levels and clinical outcomes.

Results

The mean vitamin D level was 26.7±13.0 ng/mL (n=208), and approximately one-third of patients were vitamin D deficient (n=68, 32.7%). No association was found between vitamin D deficiency and the occurrence of thrombotic events. The incidence of PE was 19.1% in vitamin D deficient patients compared to 11.4% in vitamin D sufficient patients (p=0.13). Vitamin D deficiency was positively associated with ICU admission (OR 3.047, 95%CI 1.57-5.91, p=0.001) and mortality (OR 3.76, 95%CI 1.29-11.01, p=0.016).

Conclusions

This study found no association between vitamin D deficiency and the occurrence of a new PE or DVT in hospitalized COVID-19 patients. Patients with vitamin D deficiency were more likely to be admitted to the ICU and had increased overall mortality.

## Introduction

The novel coronavirus disease 2019 (COVID-19) virus was identified from an outbreak in Wuhan, China, in December 2019 and quickly spread across the globe due to its high infectivity [1]. The COVID-19 pandemic has pushed many hospitals to a breaking point and continues to be a health emergency affecting nearly all nations. For several decades, the *Coronaviridae *family has been a known cause of mild to severe respiratory illness. The immunogenic spike glycoproteins present in COVID-19 have a notable affinity for the angiotensin-converting-enzyme-2 (ACE-2) receptor, which is expressed on the surface of alveolar epithelial type II, cardiac, renal, intestinal, and epithelial cells. This feature provides insight into COVID-19's ability to infiltrate and impact a multitude of organs. While COVID-19 infection may present with mild symptoms like fever and dry cough, it can also cause pneumonia, cytokine storm, and acute respiratory distress syndrome (ARDS); resulting in multiorgan failure and death. [2].

Risk factors for severity have been associated with the male sex, older age, and comorbidities such as hypertension and cardiovascular disease [3,4]. The pro-inflammatory features of chronic diseases may explain their association with severe COVID-19 and poorer outcomes. COVID-19 has had a disproportional impact on racial and ethnic minorities in the United States, and thrombotic complications have been suggested as a mediator of these disparities [5]. Despite prophylactic anti-coagulation, COVID-19 patients admitted to the intensive care unit (ICU) suffer from venous and arterial thromboembolism at high rates, with pulmonary embolism (PE) being the most frequent [6]. The hypercoagulable state of severe COVID-19 is evident by the steep increase of inflammatory markers, including D-dimer, C-reactive protein, and ferritin [7].

Vitamin D is a steroid hormone responsible for the central regulation of calcium homeostasis and bone metabolism. Vitamin D supplementation has an excellent safety profile and has been shown to protect against respiratory infections [8,9]. Additionally, vitamin D is a potent immunomodulator with antimicrobial and anti-inflammatory properties. Vitamin D suppresses inflammation by inhibiting T helper-1 cell cytokine production, which may help prevent the cytokine storm syndrome associated with severe COVID-19 [10]. Therefore, one hypothesis argues that vitamin D (cholecalciferol) could have protective and therapeutic effects against COVID-19. The anti-thrombogenic effects of vitamin D are achieved through the upregulation of antithrombin and thrombomodulin and the downregulation of critical prothrombotic factors like tissue factors. Enhanced thrombogenicity in vitamin D -receptor-deficient mice support the importance of vitamin D signaling in maintaining antithrombotic homeostasis [11]. Low serum vitamin D is an independent predictor of deep vein thrombosis (DVT) in patients recovering from an ischemic stroke and within the general population [12,13].

The role of vitamin D in COVID-19 remains controversial; however, an association between vitamin D deficiency and poor COVID-19 outcomes continues to appear in the literature [14]. Merzon et al. identified low vitamin D levels as an independent risk factor for COVID-19 infection and hospitalization [15], and a meta-analysis of hospitalized COVID-19 patients illustrated that supplementation with vitamin D was associated with reduced admission rates to the ICU [16]. However, some investigations have failed to elucidate the importance of vitamin D levels in COVID-19 risk [17].

Community-level socioeconomic factors play a critical role in COVID-19 disease incidence and mortality. Area Deprivation Index (ADI) measures neighborhood socioeconomic disadvantage based on income, education, employment, and housing quality. Many lifestyle-related behavioral factors such as tobacco use, poor diet, and physical inactivity, are influenced by social factors like socioeconomic status [18]. Living in a disadvantaged neighborhood is associated with a higher risk of having chronic medical conditions independent of age, sex, race, ethnicity, and level of education [19]. A patient's ADI represents the influence of neighborhood safety, access to food, stress, and health behaviors. These are critical social determinants of health that may impact the risk for vitamin D deficiency and severe COVID-19 disease.

Vitamin D is a safe and widely available supplement that can potentially be therapeutic and protective in patients with COVID-19. In this study, we evaluated vitamin D levels in hospitalized COVID-19 patients who were screened for pulmonary embolism with computed tomography pulmonary angiogram (CTPA). The main objective was to understand the relationship between vitamin D levels and the occurrence of thrombotic events (PE or DVT) in hospitalized COVID-19 patients, as well as other clinical outcomes such as ICU requirement, mechanical ventilation, and mortality.

This article was previously presented as an abstract and poster at the American Thoracic Society 2022 International Conference at San Francisco, California, United States, on May 17, 2022.

## Materials and methods

Study design and participants

**Figure 1:**
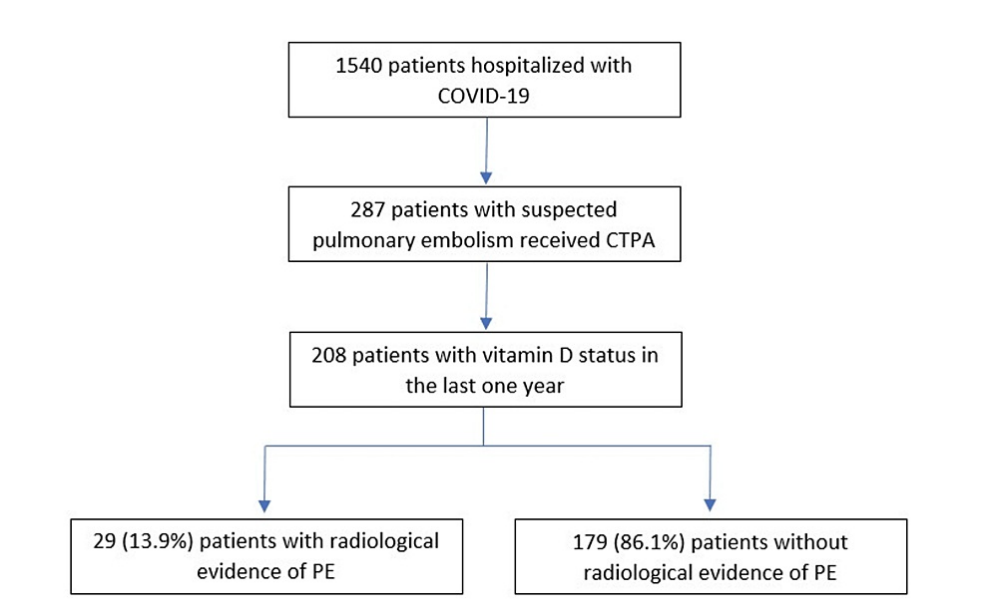
Flow chart depicting inclusion criteria Adult patients (≥18 years of age) with a confirmed COVID-19 diagnosis who had a CTPA to rule out PE during their hospitalization and a documented vitamin D level in the past year were included. COVID-19: coronavirus disease 2019; CTPA: computed tomography pulmonary angiography; PE: pulmonary embolism

This was a retrospective, cross-sectional study among consecutively hospitalized COVID-19 patients from January 1, 2020, to February 5, 2021, who underwent a CTPA based on clinical suspicion of PE (Figure 1). The inclusion criteria were: hospitalized patients who received a CTPA, had a positive COVID-19 PCR result during hospitalization, and had vitamin D levels available during their hospitalization or within the past year. This study was approved by the University of South Florida Institutional Review Board Committee (approval number: 2035).

Data collection

A manual chart review of patient records was performed to identify patients that received a CTPA due to clinical suspicion of PE and had a positive COVID-19 polymerase chain reaction (PCR) test during their hospitalization. Relevant medical history, demographics, inflammatory marker levels, vitamin D levels, and hospital course parameters were collected.

Demographics

Demographic variables included patient age, gender, race, ethnicity, body mass index (BMI), and ADI. To obtain the ADI, patient zip codes were entered into the Neighborhood Atlas® (2018, ADI version 3; University of Wisconsin School of Medicine and Public Health, Madison, Wisconsin, United States; downloaded from: https://www.neighborhoodatlas.medicine.wisc.edu/). The ADI is stratified into 10 state-specific ranking deciles ranging from least disadvantaged (1) to most disadvantaged (10). In this study, ADI is described as low area deprivation (deciles 1-3), medium area deprivation (deciles 4-7), and high area deprivation (deciles 8-10).

Definition of medical comorbidities

Immunocompromised was defined as immunosuppressing conditions such as HIV or medication such as chronic steroids, chemotherapeutic agents, and immunosuppressive antibody treatments. History of hypercoagulable state was defined as a history of DVT, PE, lupus anticoagulant, stroke or transient ischemic attack, factor V Leiden, sickle cell disease, immune thrombolytic purpura, peripheral vascular disease, and cancer. Heart disease was defined as cardiovascular arterial disease or heart failure. Lung disease was defined as chronic obstructive pulmonary disease, asthma, pulmonary fibrosis, interstitial lung disease, lung transplant, lung cancer, or pulmonary hypertension. Liver disease was defined as cirrhosis or hepatitis. History of anti-coagulation was defined as the use of rivaroxaban, apixaban, warfarin, dabigatran, low molecular weight heparin (enoxaparin), heparin, aspirin, and other anti-coagulation or antiplatelet therapies. History of cancer was defined as both active and recovered status of cancer. Other comorbidity variables included hypertension, diabetes, and atrial fibrillation.

Definition of hospital interventions

Hospital vitamin D supplementation was defined as any dose or form of vitamin D given during hospitalization. ICU and hospital length of stay were calculated by adding the total number of days spent in the hospital or ICU across all COVID-19-related hospitalizations. Hospital readmission was defined as a second hospitalization for COVID-19-related symptoms. COVID-19 treatment was defined as administering remdesivir, tociluzimab, convalescent plasma, bamlanivimab, hydroxychloroquine, REGEN-COV (casirivimab and imdevimab), dexamethasone, or other steroids. Hospital anti-coagulation was defined as the administration of rivaroxaban, apixaban, warfarin, dabigatran, low molecular weight heparin (enoxaparin), unfractionated heparin, aspirin, and other anti-coagulation or antiplatelet therapies during the hospitalization. Mortality was defined as in-hospital death or death after discharge from the hospital.

Laboratory values

Vitamin D deficiency was defined as <20 ng/mL. Peak values for inflammatory markers (D-dimer, fibrinogen, ferritin, CRP, and lactate dehydrogenase (LDH)) were defined as the highest value recorded during initial hospitalization or subsequent readmissions.

Outcomes

The primary outcomes of this study were the development of new PE or DVT during hospitalization, admission to the ICU, and mortality. Secondary outcome variables collected include the length of hospital stay, the length of ICU stay, hospital readmission, and administration of oxygen or mechanical ventilation.

Statistical methods

The data are summarized as frequencies and proportions for categorical variables and descriptive statistics, including mean and standard deviation for continuous variables. Receiver operating characteristic (ROC) curve analysis was used to determine the optimal cutoff for vitamin D deficiency. Differences between groups, those deficient in vitamin D and those not, were assessed using Chi-square tests for categorical variables, and Mann-Whitney U tests for continuous variables. DVT/PE, ICU admission, and mortality outcomes were assessed with each predictor variable. Final multivariable models were based on statistically significant predictors in univariate analyses, reduction of multicollinearity, and sufficiency of available data. Binary logistic regression models for ICU admission, mortality, and thrombosis assessed the relationship of vitamin D deficiency, with these outcomes adjusting for all significant univariate predictors. Findings are presented as odds ratio (OR) and 95% confidence interval (CI). All statistical tests used a significance level of 0.05. Analysis was completed using IBM SPSS Statistics for Windows, Version 27.0 (Released 2020; IBM Corp., Armonk, New York, United States).

## Results

Patient characteristics

Between January 1, 2020, and February 5, 2021, 1540 patients were diagnosed with COVID-19 in the Tampa General Hospital and University of South Florida healthcare systems. Among these patients, 287 (18.6%) had a CTPA performed due to suspicion of PE, of which 208 also had vitamin D levels available and were included in the final analysis. There were 109 males (52.4%) and 99 females (47.6%). The mean age was 59 (SD 14.8) years, and the mean BMI was 31.5 (SD 8.9). The distribution of race was 93 (44.7%) White, 55 (26.4%) Black, and 57 (27.4%) others. The demographic, epidemiological, and clinical characteristics of the study population are summarized in Table 1.

**Table 1:** Demographics and covariates by vitamin D status ADI: Area Deprivation Index; DVT: deep vein thrombosis; PE: pulmonary embolism; COVID-19: coronavirus disease 2019; CRP: C-reactive protein; LDH: lactate dehydrogenase

Variable		Vitamin D ≥ 20 ng/ml (N=140), N (%)	Vitamin D < 20 ng/ml (N=68), N (%)	P-value
Demographics				
Age (years)		58.9 ± 14.7	59.4 ± 15.1	0.984
Gender				0.196
	Male	69 (49.3)	40 (58.8)	
	Female	71 (50.7)	28 (41.2)	
Race				<0.001
	White	75 (53.6)	18 (27.7)	
	African American	27 (19.3)	28 (43.1)	
	Other	38 (27.1)	19 (29.2)	
Ethnicity				0.422
	Not Hispanic/Latino	105 (75.0)	46 (69.7)	
	Hispanic/Latino	35 (25.0)	20 (30.3)	
ADI				0.049
	Low area deprivation	32 (23.0)	6 (9.1)	
	Medium area deprivation	58 (41.7)	30 (45.5)	
	High area deprivation	49 (35.3)	30 (45.5)	
Comorbidities				
BMI (kg/m^2^)		31.21 ± 14.74	32.17 ± 10.57	0.823
Hypertension		90 (64.3)	46 (67.6)	0.633
Diabetes		53 (37.9)	29 (42.6)	0.507
Heart Disease		39 (27.9)	17 (25.0)	0.663
Immunocompromised		25 (17.9)	7 (10.3)	0.156
Lung Disease		33 (23.6)	21 (30.9)	0.259
Liver Disease		9 (6.4)	4 (5.9)	1.000
Atrial Fibrillation		8 (5.7)	6 (8.8)	0.393
Hypercoagulable State		20 (14.3)	12 (17.6)	0.529
History of DVT		6 (4.3)	9 (13.2)	0.041
History of PE		7 (5.0)	3 (4.4)	1.000
History of Cancer		24 (17.1)	4 (5.9)	0.026
History of Anti-coagulation		48 (34.3)	19 (27.9)	0.358
Hospital Course				
Hospital Vitamin D Supplementation		45 (32.1)	41 (60.3)	<0.001
Hospital Anti-coagulation		132 (94.3)	63 (92.6)	0.761
COVID-19 Treatment		111 (79.9)	52 (77.6)	0.710
Outcomes				
ICU Admission		51 (36.4)	40 (58.8)	0.002
ICU Length of Stay (days)		16.12 ± 18.61	13.44 ± 14.83	0.359
Hospital Length of Stay (days)		12.59 ± 16.66	14.38 ± 17.01	0.059
Hospital Readmission		25 (17.9)	17 (25.0)	0.229
Oxygen Administration		114 (81.4)	59 (86.8)	0.335
Mechanical Ventilation		23 (16.4)	13 (19.1)	0.631
DVT		12 (8.6)	8 (11.8)	0.464
PE		16 (11.4)	13 (19.1)	0.133
DVT or PE		25 (17.9)	19 (27.9)	0.095
Overall Mortality		10 (14.7)	8 (5.7)	0.030
Laboratory Test Results				
Peak D-dimer (μg/mL)		7.97 ± 16.12	12.47 ± 23.95	0.058
Peak Fibrinogen (mg/dL)		651.32 ± 199.34	647.38 ± 249.19	0.852
Peak Ferritin (ng/mL)		2479.92 ± 5006.88	2984.49 ± 5734.98	0.761
Peak CRP (mg/dL)		14.32 ± 10.26	16.73 ± 11.46	0.185
Peak LDH (U/L)		688.64 ± 850.14	564.25 ± 239.64	0.358

Vitamin D deficiency status

**Figure 2:**
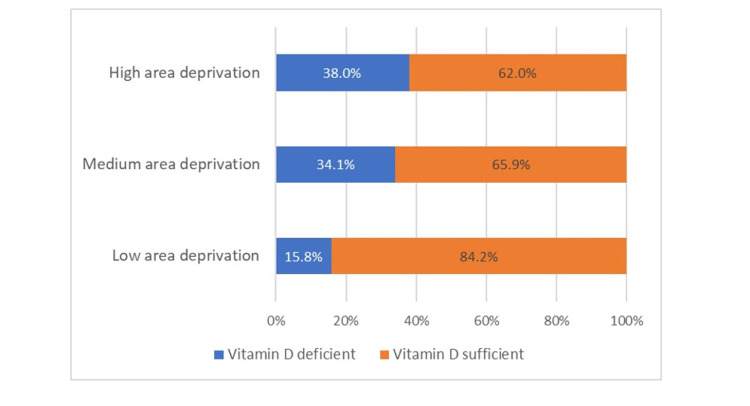
Vitamin D distribution across ADI Vitamin D deficient: blue, <20 ng/ml; Vitamin D sufficient: orange, ≥ 20 ng/ml Total patients living in high area deprivation = 79; Total patients living in medium area deprivation = 88; Total patients living in low area deprivation = 38. ADI: Area Deprivation Index

Approximately one-third of patients were categorized as deficient with vitamin D levels <20 ng/mL (n=68, 32.7%). The mean vitamin D level on hospital admission was 26.7 ng/ml (SD 12.97). After including vitamin D insufficiency (<30 ng/mL), the prevalence was 63.9%, resulting in only 35.1% with sufficient levels of vitamin D (≥30 ng/mL). Vitamin D deficiency was significantly more likely among African American patients (27.7% among White vs 43.1% among African Americans, p<0.001) and patients living in an area of high deprivation (p=0.049). Figure 2 shows the distribution of patients who were vitamin D deficient and sufficient based on ADI. Patients were more likely to be vitamin D deficient if they had a history of DVT (p=0.041). Patients were more likely to be vitamin D sufficient if they had a history of cancer (p=0.026). Fifty-six (26.9%) patients were prescribed vitamin D supplementation before their hospitalization, and 28 (13.5%) had a documented history of vitamin D deficiency.

Hospital course

Within this cohort, 195 (93.8%) patients received anti-coagulation therapy during hospitalization. The number of patients who received vitamin D supplementation was 86 (41.3%). The overall mortality was 18 (8.7%) patients. A total of 173 (83%) patients received oxygen, and 91 (43.8%) were admitted to the ICU. 17.3% (36) of patients were intubated and required mechanical ventilation. The average length of hospital stay was thirteen days and the average length of ICU stay was fifteen days. 20.2% (42) of patients were readmitted to the hospital at least once.

There was no significant association between vitamin D level and the use of mechanical ventilation (p=0.631), oxygen administration (p=0.335), length of hospital stay (p=0.059), length of ICU stay (p=0.359), or hospital readmission (p=0.229) as seen in Table 1.

ICU admission

In univariate analysis, vitamin D deficiency was significantly associated with ICU admission (OR 2.49, 95%CI 1.38-4.51). After adjusting for significant covariates listed in Table 2, vitamin D deficiency remained a significant predictor of ICU admission in multivariate analysis (OR 2.87, 95%CI 1.0-7.79, p=0.038). In the multivariate model, ICU admission was also significantly associated with age (OR 1.04, 95%CI 1.01-1.08, p=0.024) and length of hospital stay (OR 1.28, 95%CI 1.13-1.46, p<0.001).

**Table 2:** Univariate and multivariate factors predicting ICU admission Multivariate model assessing ICU admission adjusted for vitamin D deficiency, mortality, age, oxygen administration, hospital vitamin D supplementation, hospital length of stay, peak D-dimer, peak CRP, peak fibrinogen, and peak LDH. CRP: C-reactive protein; LDH: lactate dehydrogenase

Variable	Univariate odds ratio (95% CI)	Multivariate odds ratio (95% CI)	Multivariate model P-value
Vitamin D deficiency	2.49 (1.38-4.51)	2.84 (1.04-7.73)	0.041
Mortality	26.65 (3.47-204.5)	4.85 (0.40-58.26)	0.214
Age (years)	1.04 (1.02-1.06)	1.04 (1.003-1.074)	0.033
Oxygen administration	17.48 (4.07-75.13)	2.11 (0.31-14.54)	0.450
Hospital vitamin D supplementation	2.14 (1.22-3.75)	1.28 (0.51-3.19)	0.596
Hospital length of stay (days)	1.26 (1.17-1.37)	1.28 (1.12-1.46)	<0.001
Peak D-dimer (μg/mL)	1.05 (1.02-1.09)	0.99 (0.95-1.04)	0.848
Peak CRP (mg/dL)	1.09 (1.06-1.13)	1.04 (0.97-1.12)	0.264
Peak fibrinogen (mg/dL)	1.002 (1.001-1.004)	0.99 (0.995-1.001)	0.998
Peak LDH (U/L)	1.003 (1.001-1.004)	1.000 (0.999-1.002)	0.634

Mortality

The overall mortality in this cohort was 8.7% (18), and 16 of those deaths occurred in the hospital. Vitamin D deficiency was a significant predictor of mortality in univariate (OR 2.85, 95%CI 1.07-7.58) and multivariate analyses (OR 12.73, 95%CI 1.91-84.99) (p=0.009) (Table 3). Other predictors that showed no significant association with mortality in the multivariable analysis included ICU admission, DVT or PE, hospital LOS, fibrinogen, D-dimer, CRP, and LDH.

**Table 3:** Univariate and multivariate factors predicting mortality Multivariate model assessing mortality adjusted for vitamin D deficiency, ICU admission, DVT or PE, length of stay, peak D-dimer, peak CRP, peak fibrinogen, and peak LDH. CRP: C-reactive protein; LDH: lactate dehydrogenase; DVT: deep vein thrombosis; PE: pulmonary embolism

Variable	Univariate odds ratio (95% CI)	Multivariate odds ratio (95% CI)	Multivariate model P-value
Vitamin D deficiency	2.85 (1.07-7.58)	7.12 (1.34-37.73)	0.021
ICU admission	26.65 (3.47-204.49)	5.47 (0.50-60.50)	0.166
DVT or PE	3.42 (1.26-9.28)	1.52 (0.28-8.27)	0.625
Hospital length of stay (days)	1.03 (1.01-1.05)	0.99 (0.96-1.04)	0.967
Peak D-dimer (μg/mL)	1.05 (1.02-1.09)	1.02 (0.99-1.07)	0.240
Peak CRP (mg/dL)	1.125 (1.07-1.19)	1.08 (0.996-1.17)	0.061
Peak fibrinogen (mg/dL)	1.003 (1.001-1.006)	0.999 (0.995-1.002)	0.467
Peak LDH (U/L)	1.002 (1.001-1.003)	1.002 (1.000-1.004)	0.057

DVT and PE

In the study population, there were 20 (9.6%) cases of DVT, 29 (13.9%) cases of PE, and 44 (21.12%) cases of DVT or PE (five patients had both PE and DVT). Vitamin D level was not significantly associated with thrombosis (p=0.097). The multivariate model, including mortality, length of stay, history of PE, peak ferritin, peak D-dimer, and peak CRP showed a history of PE (OR 6.80, 95%CI 1.18-39.34, p=0.032) and peak D dimer (OR 1.08, 95%CI 1.04-1.13, p<0.001) to be significant predictors of thrombosis in this population (Table 4). There was no statistical significance when analyzing vitamin D levels and the incidence of only PE (p=0.133) or only DVT (p=0.464).

**Table 4:** Univariate and multivariate factors predicting DVT or PE Multivariate model assessing DVT or PE adjusted for mortality, hospital length of stay, peak D-dimer, and peak CRP. DVT: deep vein thrombosis; PE: pulmonary embolism; CRP: C-reactive protein

Variable	Univariate odds ratio (95% CI)	Multivariate odds ratio (95% CI)	Multivariate model P-value
Mortality	3.42 (1.26-9.28)	0.64 (0.14-2.81)	0.550
Hospital length of stay (days)	1.03 (1.02-1.05)	1.02 (0.99-1.05)	0.136
History of PE	4.08 (1.13-14.78)	6.63 (1.15-38.31)	0.035
Peak D-dimer (μg/mL)	1.10 (1.05-1.14)	1.08 (1.04-1.14)	<0.001
Peak CRP (mg/dL)	1.04 (1.01-1.07)	1.01 (0.96-1.06)	0.785

## Discussion

The importance of vitamin D levels concerning the severity of COVID-19 infection is unclear. Although many hypothesized mechanisms support a protective role for vitamin D, observational research studies have conflicting conclusions. There have not been adequate randomized controlled trials to draw a definitive conclusion. This retrospective cohort study demonstrates no significant correlation between vitamin D levels and thromboembolism (PE or DVT) rates in hospitalized COVID-19 patients screened for PE with CTPA. However, ICU admission and mortality rates were significantly higher in patients with vitamin D deficiency, and these differences remained after adjusting for confounding variables. The prevalence of vitamin D deficiency was high (32.7%). African American race and living in an area with high deprivation strongly correlated with vitamin D deficiency.

The results of this study suggest that adequate vitamin D levels may provide a mortality benefit for hospitalized COVID-19 patients. These patients had a high clinical suspicion of PE that prompted CTPA imaging, which indicates that they had acute COVID-19 symptoms and were in moderate to severe distress. Sufficient vitamin D levels (≥ 20 ng/ml) may have helped lower the probability of progressing to ARDS and requiring ICU-level care through enhanced immune regulation and antithrombotic effects. Patients with a deficiency in vitamin D may have either a weakened immune response or an uncontrolled immune response to COVID-19. This dysregulation may contribute to the development of a more severe infection. This study suggests that vitamin D's protective action against COVID-19 is multifactorial and not exclusively through preventing thrombosis. 

The population at risk for vitamin D deficiency is congruent with those who were heavily impacted by COVID-19. Interestingly, our findings did not support well-known risk factors for vitamin D deficiency, including male sex, older age, and obesity [20]. However, there was a higher prevalence of vitamin D deficiency in patients with low socioeconomic status living in disadvantaged neighborhoods. COVID-19 has disproportionately affected racial and ethnic minority groups in the United States, and thrombotic complications have been suggested as a mediator of these disparities [21]. Studies have shown an increased incidence of DVT/PE in African American COVID-19 patients, which was not seen in this study [22]. Instead, our study supports vitamin D levels as a more recognizable mediator between worse COVID-19 outcomes and the racial and ethnic populations more likely to reside in highly deprived neighborhoods.

Vitamin D has been hypothesized to play a key role in reducing coagulation abnormalities in COVID-19 patients [23]. However, observational studies have reported conflicting results on the relationship between vitamin D and thrombosis. Also, the majority of these studies have not factored in patient-level social risk factors such as socioeconomic status in their analyses [12,13,24,25].

Lohia et al. found no association between vitamin D levels and clinical outcomes, including developing a new PE or DVT. However, their cohort had very few patients with DVT or PE [24]. Having participants selected based on CTPA screening for PE in our study helped secure a moderate number of PE cases for statistical analysis. Although research supports an anti-thrombogenic role of vitamin D, this effect may not be robust enough to protect against developing a PE or DVT in COVID-19 infection based on our results. The investigated cohort of this study displayed a positive correlation between lower vitamin D levels and a history of DVT. This connection offers insight into the potential importance of sufficient vitamin D in thrombosis prevention.

This increased mortality of patients with vitamin D deficiency in this study is contrary to other studies showing increased hospitalization risk but no excess mortality risk [26]. Hernandez et al. reported lower mean vitamin D levels in COVID-19 patients than controls. Still, they found no relationship between vitamin D deficiency and the severity of illness like ICU admission, mechanical ventilation, or mortality [25]. A recent meta-analysis by D'Ecclesiis et al. found a significant reduction in mortality risk and severity of COVID-19 disease in those taking vitamin D supplementation. This meta-analysis also indicated a twofold increase in severity and mortality risk for COVID-19 patients with low vitamin D levels [27].

African American race and socioeconomic factors have been reported as risk factors for vitamin D deficiency. Forrest et al. identified that being African American or Hispanic, having no college education, being in poor health status, being obese, having low HDL cholesterol, and not consuming milk products daily are all independent risk factors for vitamin D deficiency [28].

The ADI represents a composite score of indicators such as median family income, unemployment rate, family poverty rate, single-parent household rate, median home value, and household crowding [29]. Living in the most disadvantaged neighborhoods has been shown to predict COVID-19 mortality independent of race, age, and sex [30]. Areas with socioeconomic inequalities likely have reduced access to healthcare and healthy foods, which can explain the higher rates of vitamin D deficiency in these disadvantaged communities.

A high prevalence of vitamin D deficiency in areas with a high ADI was expected since these areas are associated with reduced access to nutritious foods due to poverty, low education, and the availability of supermarkets. These populations also are impacted by many factors detrimental to health, like crime, substance use, unsanitary living conditions, and poor access to healthcare. Although ADI did not have a significant statistical association with any clinical outcomes, complications resulting from living in a deprived neighborhood could have contributed to more significant mortality and ICU admission rates in vitamin D-deficient patients.

At severely low levels of vitamin D (< 10ng/ml), the loss of antithrombotic properties of vitamin D signaling can lead to an increased risk of thromboembolism formation. However, there was not a substantial number of participants in this study with vitamin D levels below 10 ng/ml to draw any conclusions. Vitamin D's role in calcium absorption may also factor into blood coagulation. Calcium is an important ion in the coagulation cascade. Therefore, lower serum-free ionized calcium levels may offer some anti-coagulation effects that help offset low vitamin D levels [31]. The indications for vitamin D supplementation include low vitamin D levels, current vitamin D supplementation, and a history of vitamin D deficiency. Most patients who were found to be vitamin D deficient on admission were given vitamin D supplementation. This intervention may underly the similar rates of PE and DVT in patients with lower vitamin D levels. Since vitamin D is an inexpensive and safe supplement, there is a valid argument for providers to initiate supplementation in high-risk patients who present with vitamin D deficiency and COVID-19 or another viral upper respiratory infection.

Several limitations of the study need to be acknowledged. Patients were given different doses of vitamin D supplementation in the hospital, and the effects of these differences were not analyzed. Patients who may have been taking vitamin D supplementation without a prescription were not accounted for. Peak levels of inflammatory markers were used for this analysis. However, using the average level over the hospital stay would be a more accurate representation of inflammatory activity. The analysis did not include smoking status or a family history of DVT, PE, or clotting disorders and may serve as a confounding variable. Since active malignancy is associated with a hypercoagulable state, the seven patients with active cancer should have been separated from the twenty-one patients in remission. Many patients were excluded from the study due to missing data on vitamin D levels. This study would be more accurate if all vitamin D levels were from the time of infection rather than having some levels from up to one year before infection. However, the majority of vitamin D levels were obtained at the time of hospital admission. Although the entire study population was screened for PE, not everyone was screened for DVT with Doppler ultrasound, so the incidence of DVT is likely higher than recorded. Since this study population includes only patients screened for PE with CTPA, ICU admission and mortality outcomes have low generalizability to patients with a lower acuity COVID-19 infection.

Exploring the social and biological mediators of race and ethnic disparities in COVID-19 can inspire the development of improved preventive treatment strategies, allocation of resources, and values-based patient-centered care. This study supports the need for prospective clinical trials to determine whether replenishing vitamin D levels is a beneficial public health strategy that will reduce the severity of COVID-19 diseases. The ADI index is a powerful tool that can direct public health initiatives by identifying populations with a high risk of nutritional deficiencies.

## Conclusions

This study found a significant association between low vitamin D levels and increased mortality and ICU requirement in hospitalized patients with COVID-19 screened for PE. Vitamin D level was not associated with thromboembolism (PE or DVT). Further studies are warranted to explore the role of vitamin D and the clinical course of COVID-19 infection. Increased screening and supplementation for vitamin D deficiency, especially in disadvantaged neighborhoods, may be an effective strategy to reduce the risk of developing a life-threatening COVID-19 infection. This study furthers the understanding of vitamin D's impact on thromboembolic events and clinical outcomes in patients with COVID-19.
